# Effectiveness of a Third Dose of mRNA Vaccines Against COVID-19–Associated Emergency Department and Urgent Care Encounters and Hospitalizations Among Adults During Periods of Delta and Omicron Variant Predominance — VISION Network, 10 States, August 2021–January 2022

**DOI:** 10.15585/mmwr.mm7104e3

**Published:** 2022-01-21

**Authors:** Mark G. Thompson, Karthik Natarajan, Stephanie A. Irving, Elizabeth A. Rowley, Eric P. Griggs, Manjusha Gaglani, Nicola P. Klein, Shaun J. Grannis, Malini B. DeSilva, Edward Stenehjem, Sarah E. Reese, Monica Dickerson, Allison L. Naleway, Jungmi Han, Deepika Konatham, Charlene McEvoy, Suchitra Rao, Brian E. Dixon, Kristin Dascomb, Ned Lewis, Matthew E. Levy, Palak Patel, I-Chia Liao, Anupam B. Kharbanda, Michelle A. Barron, William F. Fadel, Nancy Grisel, Kristin Goddard, Duck-Hye Yang, Mehiret H. Wondimu, Kempapura Murthy, Nimish R. Valvi, Julie Arndorfer, Bruce Fireman, Margaret M. Dunne, Peter Embi, Eduardo Azziz-Baumgartner, Ousseny Zerbo, Catherine H. Bozio, Sue Reynolds, Jill Ferdinands, Jeremiah Williams, Ruth Link-Gelles, Stephanie J. Schrag, Jennifer R. Verani, Sarah Ball, Toan C. Ong

**Affiliations:** ^1^CDC COVID-19 Response Team; ^2^Department of Biomedical Informatics, Columbia University Irving Medical Center, New York, New York; ^3 ^New York Presbyterian Hospital, New York, New York; ^4^Center for Health Research, Kaiser Permanente Northwest, Portland, Oregon; ^5^Westat, Rockville, Maryland; ^6^Baylor Scott & White Health, Temple, Texas; ^7^Texas A&M University College of Medicine, Temple, Texas; ^8^Kaiser Permanente Vaccine Study Center, Kaiser Permanente Northern California, Oakland, California; ^9^Center for Biomedical Informatics, Regenstrief Institute, Indianapolis, Indiana; ^10^Indiana University School of Medicine, Indianapolis, Indiana; ^11^HealthPartners Institute, Minneapolis, Minnesota; ^12^Division of Infectious Diseases and Clinical Epidemiology, Intermountain Healthcare, Salt Lake City, Utah; ^13^Department of Medicine, University of Colorado, Anschutz Medical Campus, Aurora, Colorado; ^14^Fairbanks School of Public Health, Indiana University, Indianapolis, Indiana; ^15^Children's Minnesota, Minneapolis, Minnesota; ^16^Vanderbilt University, Nashville, Tennessee.

Estimates of COVID-19 mRNA vaccine effectiveness (VE) have declined in recent months (*1*,*2*) because of waning vaccine induced immunity over time,* possible increased immune evasion by SARS-CoV-2 variants (*3*), or a combination of these and other factors. CDC recommends that all persons aged ≥12 years receive a third dose (booster) of an mRNA vaccine ≥5 months after receipt of the second mRNA vaccine dose and that immunocompromised individuals receive a third primary dose.^†^ A third dose of BNT162b2 (Pfizer-BioNTech) COVID-19 vaccine increases neutralizing antibody levels (*4*), and three recent studies from Israel have shown improved effectiveness of a third dose in preventing COVID-19 associated with infections with the SARS-CoV-2 B.1.617.2 (Delta) variant (*5*–*7*). Yet, data are limited on the real-world effectiveness of third doses of COVID-19 mRNA vaccine in the United States, especially since the SARS-CoV-2 B.1.1.529 (Omicron) variant became predominant in mid-December 2021. The VISION Network^§^ examined VE by analyzing 222,772 encounters from 383 emergency departments (EDs) and urgent care (UC) clinics and 87,904 hospitalizations from 259 hospitals among adults aged ≥18 years across 10 states from August 26, 2021^¶^ to January 5, 2022. Analyses were stratified by the period before and after the Omicron variant became the predominant strain (>50% of sequenced viruses) at each study site. During the period of Delta predominance across study sites in the United States (August–mid-December 2021), VE against laboratory-confirmed COVID-19–associated ED and UC encounters was 86% 14–179 days after dose 2, 76% ≥180 days after dose 2, and 94% ≥14 days after dose 3. Estimates of VE for the same intervals after vaccination during Omicron variant predominance were 52%, 38%, and 82%, respectively. During the period of Delta variant predominance, VE against laboratory-confirmed COVID-19–associated hospitalizations was 90% 14–179 days after dose 2, 81% ≥180 days after dose 2, and 94% ≥14 days after dose 3. During Omicron variant predominance, VE estimates for the same intervals after vaccination were 81%, 57%, and 90%, respectively. The highest estimates of VE against COVID-19–associated ED and UC encounters or hospitalizations during both Delta- and Omicron-predominant periods were among adults who received a third dose of mRNA vaccine. All unvaccinated persons should get vaccinated as soon as possible. All adults who have received mRNA vaccines during their primary COVID-19 vaccination series should receive a third dose when eligible, and eligible persons should stay up to date with COVID-19 vaccinations.

VISION Network methods have been previously published (*1*,*8*,*9*). In brief, eligible medical encounters were defined as those among adults aged ≥18 years with a COVID-19–like illness diagnosis** who had received molecular testing (primarily reverse transcription–polymerase chain reaction assay) for SARS-CoV-2 (the virus that causes COVID-19) within 14 days before or 72 hours after the admission or encounter. The study period began on August 26, 2021, 14 days after the first U.S. recommendation for a third mRNA COVID-19 primary vaccine dose in immunocompromised persons.^††^ The date when the Omicron variant became predominant was determined for each study site based on state and national surveillance data. Recipients of Ad26.COV2 (Janssen [Johnson & Johnson]), 1 or >3 doses of an mRNA vaccine, and those for whom 1–13 days had elapsed since receipt of any dose were excluded. Immunocompromised patients were identified by previously published diagnosis codes^§§^ (*9*).

VE was estimated using a test-negative design, comparing the odds of a positive SARS-CoV-2 test result between vaccinated and unvaccinated patients using multivariable logistic regression models, as previously described^¶¶^ (*1*,*8*,*9*). Vaccination status was categorized based on the number of vaccine doses received and number of days from vaccination to the index medical encounter date.*** Potential effect modification by vaccine product, age group (aged 18–64 years versus ≥65 years), and immunocompromised status (for whom a third dose is the last dose in their primary series) was assessed by adding interaction terms for vaccination by these covariates to the regression model. Effect modification was only examined for medical encounters during the Delta period of predominance, given relatively sparse data during the Omicron period. A statistically significant difference was indicated by a p-value <0.01 for an interaction term, 95% CI that did not overlap, or standardized mean or proportion differences ≥0.2 indicating nonnegligible difference in distributions of vaccination or infection status (*9*). This study was reviewed and approved by the institutional review boards at participating sites or under a reliance agreement with the Westat, Inc. institutional review board.^†††^

Among 222,772 eligible ED or UC encounters, 204,745 (92%) and 18,027 (8%) occurred during the Delta- and Omicron-predominant periods, respectively (Table 1). A higher percentage of Hispanic, non-Hispanic Black, persons of unknown race/ethnicity, and adults aged <65 years were unvaccinated or had not received a third COVID-19 vaccine dose; adults aged <65 years were more likely to have received a positive SARS-CoV-2 test result. Among persons with COVID-19–like illness seeking care at ED or UC facilities who had received the second dose <180 days earlier and ≥180 days earlier, the median interval since receipt of the second dose was 137 days and 223 days, respectively. Among those who had received the third dose ≥14 days earlier, the median interval since receipt of that dose was 44 days. During the Delta-predominant period, VE against laboratory-confirmed COVID-19–associated ED and UC encounters was significantly lower among patients who had received the second vaccine dose ≥180 days earlier (76%) than it was among those who had received the dose 14–179 days earlier (86%); VE among those who had received the third mRNA COVID-19 vaccine dose was 94% (Table 2). VE after receipt of the third dose was lower among the 4% of patients with immunocompromised status (74%; 95% CI = 65%–80%) versus those without (95%; 95% CI = 94%–95%) (p<0.001) (CDC, unpublished data, 2022). During the period of Omicron predominance, the pattern was similar, although all VE estimates were significantly lower. VE against COVID-19–associated ED and UC encounters 14–179 days after receipt of dose 2 was 52%, and at ≥180 days after dose 2 was 38%. VE after receipt of 3 vaccine doses among all adults was 82%.

**TABLE 1:** Characteristics of emergency department and urgent care encounters among adults with COVID-19–like illness,* by mRNA COVID-19 vaccination status^†^ and SARS-CoV-2 test result — 10 states, August 2021–January 2022^§^

Characteristic	Total no. (column %)	mRNA COVID-19 vaccination status, no. (row %)				SMD**	Positive SARS-CoV-2 test result, no. (row %)	SMD**
		Unvaccinated	2 doses (<180 days earlier)	2 doses (≥180 days earlier)	3 doses^¶^			
**All ED/UC events**	**222,772 (100)**	**105,083 (47)**	**41,375 (19)**	**57,915 (26)**	**18,399 (8)**	**—**	**53,719 (24)**	**—**
**Variant predominance period**								
B.1.617.2 (Delta)	**204,745 (92)**	98,087 (48)	39,629 (19)	52,506 (26)	14,523 (7)	0.10	47,173 (23)	0.18
B.1.1.529 (Omicron)	**18,027 (8)**	6,996 (39)	1,746 (10)	5,409 (30)	3,876 (22)		6,546 (36)	
**Sites**								
Baylor Scott & White Health	**34,143 (15)**	19,787 (58)	4,476 (13)	8,179 (24)	1,701 (5)	0.57	7,854 (23)	0.27
Columbia University	**4,608 (2)**	2,500 (54)	921 (20)	1,031 (22)	156 (3)		803 (17)	
HealthPartners	**5,347 (2)**	1,536 (29)	1,832 (34)	1,689 (32)	290 (5)		873 (16)	
Intermountain Healthcare	**62,740 (28)**	28,837 (46)	12,210 (19)	16,615 (26)	5,078 (8)		15,593 (25)	
Kaiser Permanente Northern California	**42,919 (19)**	11,027 (26)	9,418 (22)	15,341 (36)	7,133 (17)		8,564 (20)	
Kaiser Permanente Northwest	**15,813 (7)**	6,028 (38)	4,168 (26)	4,075 (26)	1,542 (10)		2,958 (19)	
Regenstrief Institute	**35,233 (16)**	23,110 (66)	4,986 (14)	5,895 (17)	1,242 (4)		12,174 (35)	
University of Colorado	**21,969 (10)**	12,258 (56)	3,364 (15)	5,090 (23)	1,257 (6)		4,900 (22)	
**Age group, yrs**								
18–49	**117,000 (53)**	68,469 (59)	23,268 (20)	21,492 (18)	3,771 (3)	0.56	29,494 (25)	0.20
50–64	**45,056 (20)**	19,966 (44)	9,671 (21)	12,118 (27)	3,301 (7)		12,435 (28)	
65–74	**28,858 (13)**	9,187 (32)	4,625 (16)	10,140 (35)	4,906 (17)		6,492 (22)	
75–84	**21,175 (10)**	5,103 (24)	2,699 (13)	9,033 (43)	4,340 (20)		3,723 (18)	
≥85	**10,683 (5)**	2,358 (22)	1,112 (10)	5,132 (48)	2,081 (19)		1,575 (15)	
**Sex**								
Male^††^	**90,372 (41)**	44,951 (50)	15,490 (17)	22,252 (25)	7,679 (8)	0.09	24,497 (27)	−0.13
Female	**132,400 (59)**	60,132 (45)	25,885 (20)	35,663 (27)	10,720 (8)		29,222 (22)	
**Race/Ethnicity**								
White, non-Hispanic	**140,967 (63)**	63,794 (45)	25,190 (18)	38,952 (28)	13,031 (9)	0.25	33,054 (23)	0.10
Black, non-Hispanic	**21,020 (9)**	12,294 (58)	3,864 (18)	3,853 (18)	1,009 (5)		5,241 (25)	
Hispanic	**34,791 (16)**	17,426 (50)	7,147 (21)	8,130 (23)	2,088 (6)		8,765 (25)	
Other, non-Hispanic^§§^	**13,469 (6)**	4,266 (32)	3,041 (23)	4425 (33)	1,737 (13)		2,790 (21)	
Unknown	**12,525 (6)**	7,303 (58)	2,133 (17)	2,555 (20)	534 (4)		3,869 (31)	
**Chronic respiratory condition^¶¶^**								
Yes^††^	**42,449 (19)**	19,111 (45)	7,333 (17)	11,875 (28)	4,130 (10)	–0.04	8,110 (19)	0.14
No	**180,323 (81)**	85,972 (48)	34,042 (19)	46,040 (26)	14,269 (8)		45,609 (25)	
Chronic nonrespiratory condition***								
Yes^††^	**59,223 (27)**	25,123 (42)	10,380 (18)	17,654 (30)	6,066 (10)	–0.12	12,232 (21)	0.12
No	**163,549 (73)**	79,960 (49)	30,995 (19)	40,261 (25)	12,333 (8)		41,487 (25)	
**Vaccine product**								
Moderna	**44,287 (38)**	—	14,227 (32)	24,489 (55)	5,571 (13)	—	4,464 (10)	0.14
Pfizer-BioNTech	**72,307 (61)**	—	27,045 (37)	33,344 (46)	11,918 (16)		9,244 (13)	
Combination of mRNA products	**1,095 (1)**	—	103 (9)	82 (7)	910 (83)		71 (6)	

**Abbreviations:** ED = emergency department; SMD = standardized mean or proportion difference; UC = urgent care.

* Medical events with a discharge code consistent with COVID-19–like illness were included. COVID-19–like illness diagnoses included acute respiratory illness (e.g., COVID-19, respiratory failure, or pneumonia) or related signs or symptoms (cough, fever, dyspnea, vomiting, or diarrhea) using diagnosis codes from the *International Classification of Diseases, Ninth Revision* and *International Classification of Diseases, Tenth Revision*. Clinician-ordered molecular assays (e.g., real-time reverse transcription–polymerase chain reaction) for SARS-CoV-2 occurring ≤14 days before to <72 hours after the encounter date were included. Recipients of Janssen, 1 or >3 doses of an mRNA vaccine, and those for whom 1–13 days had elapsed since receipt of any dose were excluded.

^†^ Vaccination was defined as having received the listed number of doses of an mRNA-based COVID-19 vaccine ≥14 days before the medical event index date, which was the date of respiratory specimen collection associated with the most recent positive or negative SARS-CoV-2 test result before the medical event or the admission date if testing only occurred after the admission.

^§^ Partners contributing data on medical events and estimated dates of Omicron predominance were in California (December 21), Colorado (December 19), Indiana (December 26), Minnesota and Wisconsin (December 25), New York (December 18), Oregon (December 24), Texas (December 16), Utah (December 24), and Washington (December 24). The study period began in September 2021 for partners located in Texas.

^¶^ The “3 doses” category includes persons who have received a third dose in their primary series or have received an additional dose following their 2-dose primary series; this includes the reduced-dosage Moderna booster.

** An absolute standardized mean or proportion difference ≥0.20 indicates a nonnegligible difference in variable distributions between medical events for vaccinated versus unvaccinated patients; single SMD calculated by averaging pairwise comparisons of each vaccinated category versus unvaccinated and separately for patients with SARS-CoV-2–positive versus SARS-CoV-2–negative test results. For example, the age SMD calculation comparing unvaccinated versus different vaccinated categories was generated by averaging the pairwise SMD calculations for unvaccinated and 2 doses (<180 days earlier), unvaccinated and 2 doses (≥180 days earlier), and unvaccinated and 3 doses.

^††^ Indicates the reference group used for standardized mean or proportion difference calculations for dichotomous variables.

^§§^ Other race includes Asian, Hawaiian or Other Pacific islander, American Indian or Alaska Native, Other not listed, and multiple races.

^¶¶^ Chronic respiratory condition was defined as the presence of discharge code for asthma, chronic obstructive pulmonary disease, or other lung disease using diagnosis codes from the *International Classification of Diseases, Ninth Revision* and the *International Classification of Diseases, Tenth Revision.*

*** Chronic nonrespiratory condition was defined as the presence of discharge code for heart failure, ischemic heart disease, hypertension, other heart disease, stroke, other cerebrovascular disease, diabetes type I or II, other diabetes, metabolic disease, clinical obesity, clinically underweight, renal disease, liver disease, blood disorder, immunosuppression, organ transplant, cancer, dementia, neurologic disorder, musculoskeletal disorder, or Down syndrome using diagnosis codes from the *International Classification of Diseases, Ninth Revision* and the *International Classification of Diseases, Tenth Revision*.

**TABLE 2:** mRNA COVID-19 vaccine effectiveness* against laboratory-confirmed COVID-19–associated^†^ emergency department and urgent care encounters and hospitalizations among adults aged ≥18 years, by number and timing of vaccine doses^§^ and vaccine product received — VISION Network, 10 states, August 2021–January 2022^¶^

Encounter/Predominant variant period/Vaccination status	Total	SARS-CoV-2 positive test result, no. (%)	VE, %* (95% CI)
**ED or UC encounters**			
**Delta predominant**			
Unvaccinated (Ref)	**98,087**	36,542 (37.2)	—
Any mRNA vaccine			
2 doses (14–179 days earlier)	**39,629**	3,269 (8.2)	86 (85–87)
2 doses (≥180 days earlier)	**52,506**	6,893 (13.1)	76 (75–77)
3 doses	**14,523**	469 (3.2)	94 (93–94)
**Omicron predominant**			
Unvaccinated (Ref)	**6,996**	3,398 (48.6)	—
Any mRNA vaccine			
2 doses (14–179 days earlier)	**1,746**	591 (33.9)	52 (46–58)
2 doses (≥180 days earlier)	**5,409**	2,037 (37.7)	38 (32–43)
3 doses	**3,876**	520 (13.4)	82 (79–84)
**Hospitalizations**			
**Delta predominant**			
Unvaccinated (Ref)	**37,400**	14,272 (38.2)	—
Any mRNA vaccine			
2 doses (14–179 days earlier)	**14,645**	895 (6.1)	90 (89–90)
2 doses (≥180 days earlier)	**26,190**	2,563 (9.8)	81 (80–82)
3 doses	**8,092**	209 (2.6)	94 (93–95)
**Omicron predominant**			
Unvaccinated (Ref)	**460**	174 (37.8)	—
Any mRNA vaccine			
2 doses (14–179 days earlier)	**115**	14 (12.2)	81 (65–90)
2 doses (≥180 days earlier)	**488**	86 (17.6)	57 (39–70)
3 doses	**514**	24 (4.7)	90 (80–94)

**Abbreviations:** ED = emergency department; Ref = reference group; UC = urgent care; VE = vaccine effectiveness.

* VE was calculated as ([1−odds ratio] x 100%), estimated using a test-negative design, adjusted for age, geographic region, calendar time (days since August 26, 2021), and local virus circulation (percentage of SARS-CoV-2–positive results from testing within the counties surrounding the facility on the date of the encounter) and weighted for inverse propensity to be vaccinated or unvaccinated (calculated separately for each VE estimate). Generalized boosted regression trees were used to estimate the propensity to be vaccinated based on sociodemographic characteristics, underlying medical conditions, and facility characteristics.

^†^ Medical events with a discharge code consistent with COVID-19–like illness were included. COVID-19–like illness diagnoses included acute respiratory illness (e.g., COVID-19, respiratory failure, or pneumonia) or related signs or symptoms (cough, fever, dyspnea, vomiting, or diarrhea) using diagnosis codes from the *International Classification of Diseases, Ninth Revision* and *International Classification of Diseases, Tenth Revision*. Clinician-ordered molecular assays (e.g., real-time reverse transcription–polymerase chain reaction) for SARS-CoV-2 occurring ≤14 days before to <72 hours after admission were included. Recipients of Janssen, 1 or >3 doses of an mRNA vaccine, and those for whom 1–13 days had elapsed since receipt of any dose were excluded.

^§^ Vaccination status was documented in electronic health records and immunization registries and was defined as having received the listed number of doses of an mRNA-based COVID-19 vaccine ≥14 days before the medical event index date. Index date was defined as the date of respiratory specimen collection associated with the most recent positive or negative SARS-CoV-2 test result before the medical event or the admission date if testing only occurred after the admission. Three-dose recipients include persons who received a third dose in their primary series or received an additional (booster) dose after their 2-dose primary series; this includes the reduced-dosage Moderna booster.

^¶^ Partners contributing data on medical events and estimated dates of Omicron predominance were in California (December 21), Colorado (December 19), Indiana (December 26), Minnesota and Wisconsin (December 25), New York (December 18), Oregon (December 24), Texas (December 16), Utah (December 24), and Washington (December 24). The study period began in September 2021 for partners located in Texas.

Among 87,904 eligible hospitalizations, 86,327 (98%) and 1,577 (2%) occurred during the Delta- and Omicron-predominant periods, respectively (Table 3). Hospitalized adults aged <65 years or who were Hispanic, non-Hispanic Black, unknown race/ethnicity, or who did not have chronic nonrespiratory medical conditions were more likely to be unvaccinated or, if vaccinated, less likely to have received a third vaccine dose. In addition, adults aged <65 years and those without chronic nonrespiratory conditions were more likely to have received a positive SARS-CoV-2 test result. Among persons hospitalized with COVID-19–like illness who had received the second mRNA COVID-19 vaccine dose <180 days earlier and ≥180 days earlier, the median interval since receipt of the second dose was 144 days and 222 days, respectively. Among those who had received the third dose ≥14 days earlier, the median interval since receipt of that dose was 41 days. During Delta predominance, VE against laboratory-confirmed COVID-19–associated hospitalization was 90% among persons who had received the second dose 14–179 days earlier, 81% among those who had received it ≥180 days earlier, and 94% among persons who had received a third dose ≥14 days earlier (Table 2). VE after receipt of the third dose was lower among the 21% of patients with immunocompromised status (83%; 95% CI = 78%–87%) versus among those without (96%; 95% CI = 95%–97%) (p = 0.001) (CDC, unpublished data, 2022). During Omicron predominance, VE against COVID-19–associated hospitalization was 81% among 2-dose recipients who had received the second dose 14–179 days earlier, 57% among those who had received it ≥180 days earlier, and 90% at ≥14 days after receipt of a third dose. VE estimates for patients who received dose 2 ≥180 days earlier significantly declined during Omicron predominance compared with estimates during Delta predominance.

**TABLE 3:** Characteristics of hospitalizations with COVID-19–like illness,* by mRNA COVID-19 vaccination status^†^ and SARS-CoV-2 test result — 10 states, August 2021–January 2022^§^

Characteristic	Total, no. (column %)	mRNA COVID-19 vaccination status, no. (row %)				SMD**	Positive SARS-CoV-2 test result, no. (row %)	SMD**
		Unvaccinated	2 doses (<180 days earlier)	2 doses (≥180 days earlier)	3 doses^¶^			
**All hospitalizations**	**87,904 (100)**	**37,860 (43)**	**14,760 (17)**	**26,678 (30)**	**8,606 (10)**	**—**	**18,237 (21)**	**—**
**Variant predominance period**								
B.1.617.2 (Delta)	**86,327 (98)**	37,400 (43)	14,645 (17)	26,190 (30)	8,092 (9)	0.08	17,939 (21)	–0.02
B.1.1.529 (Omicron)	**1,577 (2)**	460 (29)	115 (7)	488 (31)	514 (33)		298 (19)	
**Sites**								
Baylor Scott & White Health	**15,226 (17)**	7,730 (51)	2,180 (14)	4,372 (29)	944 (6)	0.56	2,462 (16)	0.51
Columbia University	**3,254 (4)**	1,372 (42)	696 (21)	975 (30)	211 (6)		331 (10)	
HealthPartners	**1,159 (1)**	265 (23)	374 (32)	450 (39)	70 (6)		133 (11)	
Intermountain Healthcare	**8435 (10)**	4283 (51)	1158 (14)	2201 (26)	793 (9)		3117 (37)	
Kaiser Permanente Northern California	**22,181 (25)**	4,891 (22)	4,691 (21)	8,591 (39)	4,008 (18)		2,716 (12)	
Kaiser Permanente Northwest	**3,879 (4)**	1,628 (42)	927 (24)	961 (25)	363 (9)		740 (19)	
Regenstrief Institute	**23,370 (27)**	12,641 (54)	3,134 (13)	6,274 (27)	1,321 (6)		6,686 (29)	
University of Colorado	**10,400 (12)**	5,050 (49)	1,600 (15)	2,854 (27)	896 (9)		2,052 (20)	
**Age group, yrs**								
18–49	**21,128 (24)**	13,609 (64)	3,980 (19)	2,780 (13)	759 (4)	0.63	5,523 (26)	0.31
50–64	**20,193 (23)**	10,204 (51)	4,407 (22)	4,407 (22)	1,175 (6)		5,132 (25)	
65–74	**19,798 (23)**	6,952 (35)	3,283 (17)	7,052 (36)	2,511 (13)		3,684 (19)	
75–84	**17,052 (19)**	4,647 (27)	2,122 (12)	7,609 (45)	2,674 (16)		2,626 (15)	
≥85	**9,733 (11)**	2,448 (25)	968 (10)	4,830 (50)	1,487 (15)		1,272 (13)	
**Sex**								
Male^††^	**39,602 (45)**	17,468 (44)	6,131 (15)	11,969 (30)	4,034 (10)	0.04	9,252 (23)	–0.14
Female	**48,302 (55)**	20,392 (42)	8,629 (18)	14,709 (30)	4,572 (9)		8,985 (19)	
**Race/Ethnicity**								
White, non-Hispanic	**56,669 (64)**	23,297 (41)	8,855 (16)	18,333 (32)	6,184 (11)	0.25	11,743 (21)	0.17
Black, non-Hispanic	**9,628 (11)**	5,026 (52)	1835 (19)	2,240 (23)	527 (5)		1,707 (18)	
Hispanic	**11,304 (13)**	5,337 (47)	2,171 (19)	2,955 (26)	841 (7)		2,585 (23)	
Other, non-Hispanic^§§^	**5,488 (6)**	1,524 (28)	1,232 (22)	1,940 (35)	792 (14)		808 (15)	
Unknown	**4,815 (5)**	2,676 (56)	667 (14)	1,210 (25)	262 (5)		1,394 (29)	
**Chronic respiratory condition^¶¶^**								
Yes^††^	**57,225 (65)**	24,037 (42)	9,549 (17)	17,693 (31)	5,946 (10)	–0.06	11,648 (20)	0.03
No	**30,679 (35)**	13,823 (45)	5,211 (17)	8,985 (29)	2,660 (9)		6,589 (21)	
Chronic nonrespiratory condition***								
Yes^††^	**74,943 (85)**	29,810 (40)	12,787 (17)	24,270 (32)	8,076 (11)	–0.32	13,924 (19)	0.30
No	**12,961 (15)**	8,050 (62)	1,973 (15)	2,408 (19)	530 (4)		4,313 (33)	
**Vaccine product**								
Moderna	**20,236 (40)**	—	5,690 (28)	11,903 (59)	2,643 (13)	—	1,294 (6)	0.15
Pfizer-BioNTech	**29,418 (59)**	—	9,023 (31)	14,740 (50)	5,655 (19)		2,480 (8)	
Combination of mRNA products	**390 (1)**	—	47 (12)	35 (9)	308 (79)		17 (4)	

**Abbreviations:** ED = emergency department; SMD = standardized mean or proportion difference; UC = urgent care.

* Medical events with a discharge code consistent with COVID-19–like illness were included. COVID-19–like illness diagnoses included acute respiratory illness (e.g., COVID-19, respiratory failure, or pneumonia) or related signs or symptoms (cough, fever, dyspnea, vomiting, or diarrhea) using diagnosis codes from the *International Classification of Diseases, Ninth Revision* and *International Classification of Diseases, Tenth Revision.* Clinician-ordered molecular assays (e.g., real-time reverse transcription–polymerase chain reaction) for SARS-CoV-2 occurring ≤14 days before to <72 hours after admission were included. Recipients of Janssen, 1 or >3 dose of an mRNA vaccine, and those for whom 1–13 days had elapsed since receipt of any dose were excluded.

^†^ Vaccination was defined as having received the listed number of doses of an mRNA-based COVID-19 vaccine ≥14 days before the medical event index date, which was the date of respiratory specimen collection associated with the most recent positive or negative SARS-CoV-2 test result before the medical event or the admission date if testing only occurred after the admission.

^§^ Partners contributing data on medical events and estimated dates of Omicron predominance were in California (December 21), Colorado (December 19), Indiana (December 26), Minnesota and Wisconsin (December 25), New York (December 18), Oregon (December 24), Texas (December 16), Utah (December 24), and Washington (December 24). The study period began in September 2021 for partners located in Texas.

^¶^ The “3 doses” category includes persons who have received a third dose in their primary series or received an additional dose following their 2-dose primary series; this includes the reduced-dosage Moderna booster.

** An absolute standardized mean or proportion difference ≥0.20 indicates a nonnegligible difference in variable distributions between medical events for vaccinated versus unvaccinated patients. First, a single SMD was calculated by averaging pairwise comparisons of each vaccinated category versus unvaccinated, and then, a second SMD was calculated separately for SARS-CoV-2–positive versus SARS-CoV-2–negative patients. For example, the age SMD calculation comparing unvaccinated versus different vaccinated categories was generated by averaging the pairwise SMD calculations for unvaccinated and 2 doses (<180 days earlier), unvaccinated and 2 doses (≥180 days earlier), and unvaccinated and 3 doses.

^††^ Indicates the reference group used for standardized mean or proportion difference calculations for dichotomous variables.

^§§^ Other race includes Asian, Hawaiian or Other Pacific islander, American Indian or Alaska Native, Other not listed, and multiple races.

^¶¶^ Chronic respiratory condition was defined as the presence of discharge code for asthma, chronic obstructive pulmonary disease, or other lung disease using diagnosis codes from the *International Classification of Diseases, Ninth Revision* and the *International Classification of Diseases, Tenth Revision.*

*** Chronic nonrespiratory condition was defined as the presence of discharge code for heart failure, ischemic heart disease, hypertension, other heart disease, stroke, other cerebrovascular disease, diabetes type I or II, other diabetes, metabolic disease, clinical obesity, clinically underweight, renal disease, liver disease, blood disorder, immunosuppression, organ transplant, cancer, dementia, neurologic disorder, musculoskeletal disorder, or Down syndrome using diagnosis codes from the *International Classification of Diseases, Ninth Revision* and the *International Classification of Diseases, Tenth Revision.*

## Discussion

In a multistate analysis of 222,772 ED and UC encounters and 87,904 hospitalizations among adults with COVID-19–like illness during August 26, 2021–January 5, 2022, estimates of VE against laboratory-confirmed COVID-19 declined during the Omicron-predominant period compared with VE during the Delta-predominant period. During both periods, VE was significantly lower among patients who received their second mRNA COVID-19 vaccine dose ≥180 days before the medical encounters compared with those vaccinated more recently. VE increased following a third dose and was highly effective during both the Delta- and Omicron-predominant periods at preventing COVID-19–associated ED and UC encounters (94% and 82%, respectively) and preventing COVID-19–associated hospitalizations (94% and 90%, respectively).

Estimates of VE for 2 doses of an mRNA vaccine were higher against COVID-19–associated hospitalizations than against COVID-19–associated ED or UC encounters, especially during the Omicron period, which is consistent with possible vaccine attenuation of severity of COVID-19 disease but was not observed in this network previously (*1*,*8*). This study also found that immunocompromised adults had lower third dose VE against COVID-19–associated ED and UC encounters and hospitalization, which is consistent with trends observed for VE following a second dose (*9*) and is consistent with recommendations for a booster dose for this group 5 months after the additional primary dose.^§§§^

The findings in this report are subject to at least seven limitations. First, VE estimates from this study do not include COVID-19-associated outpatient visits or non-medically attended COVID-19. Second, the median interval from receipt of dose 3 to medical encounters was 41–44 days; thus, the observed performance of dose 3 is limited to a relatively short period after vaccination. Third, the reasons for the decline in VE during the Omicron period are unclear; however, the drop in VE during a short period suggests increased immune evasion by the variant. Fourth, limited data during the Omicron period reduced the precision of the VE estimates and precluded tests for effect modification. Fifth, despite adjustments to balance the differences between unvaccinated and vaccinated adults, unmeasured and residual confounding (e.g., wearing a mask and close contact with persons with COVID-19) in this observational study might have biased the estimates. Sixth, genetic characterization of patients’ viruses was not available, and analyses therefore relied on dates when the Omicron variant became predominant based on surveillance data. The Omicron period of predominance in this study likely includes medical encounters associated with the Delta variant. If VE is reduced against medical care associated with Omicron variant, this study likely overestimated VE. Finally, although the facilities in this study serve heterogeneous populations in 10 states, the findings might not be generalizable to the U.S. population.

These findings underscore the importance of receiving a third dose of mRNA COVID-19 vaccine to prevent both moderately severe and severe COVID-19, especially while the Omicron variant is the predominant circulating variant and when the effectiveness of 2 doses of mRNA vaccines is significantly reduced against this variant. All unvaccinated persons should get vaccinated as soon as possible. All adults who have received mRNA vaccines during their primary COVID-19 vaccination series should receive a third dose when eligible, and eligible persons should stay up to date with COVID-19 vaccinations.

**Summary**

**What is already known about this topic?**

COVID-19 mRNA vaccine effectiveness (VE) in preventing COVID-19 might decline because of waning of vaccine-induced immunity or variant immune evasion.

**What is added by this report?**

VE was significantly higher among patients who received their second mRNA COVID-19 vaccine dose <180 days before medical encounters compared with those vaccinated ≥180 days earlier. During both Delta- and Omicron-predominant periods, receipt of a third vaccine dose was highly effective at preventing COVID-19–associated emergency department and urgent care encounters (94% and 82%, respectively) and preventing COVID-19–associated hospitalizations (94% and 90%, respectively).

**What are the implications for public health practice?**

All unvaccinated persons should start vaccination as soon as possible. All adults who have received mRNA vaccines during their primary COVID-19 vaccination series should receive a third dose when eligible, and eligible persons should stay up to date with COVID-19 vaccinations.

## Footnotes

*
https://papers.ssrn.com/sol3/papers.cfm?abstract_id=3961378

† CDC initially recommended a third dose of mRNA vaccine for all adults 6 months after receipt of the second mRNA COVID-19 vaccine dose. On January 4, 2022, CDC amended the interval to 5 months after receipt of the second dose for recipients of the BNT162b2 (Pfizer-BioNTech) vaccine. On January 7, 2022, CDC amended the interval to 5 months for recipients of the mRNA-1273 (Moderna) vaccine. CDC recommends the Pfizer-BioNTech booster at 5 months, and an additional primary dose for certain immunocompromised persons aged ≥5 years (https://www.cdc.gov/coronavirus/2019-ncov/vaccines/recommendations/immuno.html). CDC recommends the Moderna booster at 5 months. https://www.cdc.gov/media/releases/2022/s0107-moderna-booster.html

§ Funded by CDC, the VISION Network includes Baylor Scott & White Health (Texas), Columbia University Irving Medical Center (New York), HealthPartners (Minnesota and Wisconsin), Intermountain Healthcare (Utah), Kaiser Permanente Northern California (California), Kaiser Permanente Northwest (Oregon and Washington), Regenstrief Institute (Indiana), and University of Colorado (Colorado).

¶ The study period at Baylor Scott & White Health began on September 11, 2021.

** COVID-19–like illness diagnoses included acute respiratory illness (e.g., COVID-19, respiratory failure, or pneumonia) or related signs or symptoms (cough, fever, dyspnea, vomiting, or diarrhea) using diagnosis codes from the *International Classification of Diseases, Ninth Revision* and *International Classification of Diseases, Tenth Revision*.

††
https://www.fda.gov/news-events/press-announcements/coronavirus-covid-19-update-fda-authorizes-additional-vaccine-dose-certain-immunocompromised

§§ Immunocompromising conditions were derived from lists used in previous studies of large hospital-based or administrative databases and included the following conditions: 1) solid malignancies, 2) hematologic malignancies, 3) rheumatologic or inflammatory disorders, 4) other intrinsic immune conditions or immunodeficiencies, and 5) organ or stem cell transplants.

¶¶ With a test-negative design, vaccine performance is assessed by comparing the odds of antecedent vaccination among case-patients with acute laboratory-confirmed COVID-19 and control-patients without acute COVID-19. This odds ratio was adjusted for age, geographic region, calendar time (days from August 26, 2021), and local virus circulation in the community and weighted for inverse propensity to be vaccinated or unvaccinated (calculated separately for each vaccine exposure group).

*** Index test date was defined as the date of respiratory specimen collection associated with the most recent positive or negative SARS-CoV-2 test result before the hospitalization or the hospitalization date if testing only occurred after admission.

††† 45 C.F.R. part 46; 21 C.F.R. part 56.

§§§ CDC recommends that all immunocompromised persons receive a booster dose of either Pfizer-BioNTech or Moderna COVID-19 vaccines 5 months after completing their 3-dose primary series. https://www.cdc.gov/coronavirus/2019-ncov/vaccines/recommendations/immuno.html
